# Posture restrictions do not interfere in the results of canalith repositioning maneuver

**DOI:** 10.1016/S1808-8694(15)31285-4

**Published:** 2015-10-20

**Authors:** Lucinda Simoceli, Roseli Saraiva Moreira Bittar, Mário Edvin Greters

**Affiliations:** 1 Collaborating Physician, Sector of Otoneurology, HCFMUSP; 2 Assistant Physician, Sector of Otoneurology, HCFMUSP

**Keywords:** benign paroxysmal positional vertigo, Epley maneuver, dizziness

## Abstract

Benign Paroxysmal Positional Vertigo (BPPV) is a frequent cause of dizziness and despite of the excellent results with its treatment, there is some controversy about management.

**Aim:**

To assess the efficacy of Epley Maneuver with and without post-maneuver restrictions.

**Study design:**

Prospective randomized.

**Material and Method:**

Fifty patients presenting BPPV of the posterior semicircular canal, treated with Epley Maneuver and divided into two groups: study group – 23 patients – with post-maneuver restrictions, and control group – 27 patients – without post-maneuver restrictions.

**Results:**

No significant difference was found between the studied and the control group.

**Conclusion:**

Post-maneuver restrictions do not influence the efficacy of Epley Maneuver for BPPV management.

## INTRODUCTION

Benign Paroxysmal Positional Vertigo (BPPV) is defined as a vestibular syndrome of peripheral origin characterized by short and intensive episodes of vertigo, associated with predominantly horizontal-rotation nystagmus, triggered by quick change of head position^1^. In addition to the characteristics described, symptomatology of BPPV may include imbalance, sensation of light head and nausea. Crises are normally triggered by sudden changes in head position, getting up from bed or rotating around the body. The incidence varies in epidemiological studies from 11 to 64 per 100,00/year^2^, ^3^.

BPPV is one of the most common diseases of the inner ear, reported in the literature as being responsible for approximately 17% of the clinical diagnoses of dizziness^4^, ^5^. It may be found in all age ranges, but it increases with aging^6^ and in idiopathic cases, its peak of incidence is within 50 and 70 years^1^, despite the fact that it is part of the differential diagnosis of vertigo in children^7^. In a recent cross section study conducted by the Division of Otoneurology, HCFMUSP, diagnosis of BPPV amounted to 15% of the etiologies of dizziness in the population older than 65 years^8^.

In 50 to 70% of the cases, BPPV is idiopathic or primary^5^, and the second most common cause is head trauma that corresponds to 7 to 17% of cases^4^, ^6^. Vestibular neuronitis is associated with approximately 15% of the cases^6^ and Ménière's disease is present between 0.5% and 31% of BPPV^9^, ^10^. Authors such as Gross^11^, conversely, observed that approximately 5.5% of the patients with Ménière's disease presented typical BPPV of posterior semicircular canal and these cases were of difficult clinical control of positional symptomatology. Ishiyama^12^ and Lempert^13^ have recently described the association between migraine and cause of BPPV in 5% of the cases, as observed in most of the children in the study by Uneri^7^. BPPV may be associated with inner ear surgeries in 1% of the cases, and the highest risk for its development is detected in stapes surgeries (stapedectomy and stapedotomy)^14^, ^15^.

The clinical pathology substrate corresponding to BPPV was proposed by Schuknecht^16^ in 1962, which described the presence of crystals coming from utriculus macula, which are released and then adhere to the top of posterior semicircular canal (cupololithiasis). Some years later, Hall et al.^17^ proposed the canalith therapy, which comprises the increase of endolymph density caused by the presence of free suspension particles. BPPV may be originated from any semicircular canal, but the posterior canal is the most frequently affected in the majority of cases. The natural clinical course of BPPV is self-limited and takes from weeks to months, and it normally does not respond to antivertigo drugs^18^, ^19^, ^20^. Advocated treatments are various: maneuvers of canalith repositioning - Epley maneuver is the most common one^21^, liberatory maneuvers, Semont^22^ maneuver, vestibular habituation training^23^, ^24^, ^25^, and surgical treatments such as singular neurectomy or occlusion of posterior semicircular canal that are reserved to cases non-responsive to clinical treatment^26^, ^27^.

In its first description of the maneuver of the posterior semicircular canal reposition, Epley^21^ recommended the use of bone vibration, care with posture restriction and head movement after treatment. These guidelines intended to prevent the return of the repositioned particles to the released semicircular canals. Among them, there are recommendations to sleep sitting up or with head inclination of 45^º^ (reclined armchair or the use of 2 pillows) for 48 hours after the maneuver. Some studies^5^, ^28^ recommend elevated lying down position after the maneuver for 7 days, period in which the patient should avoid sudden movements and those that trigger dizziness, not to sleep over the affected ear, and not to look up or down^29^. Some authors suggest that patients should wear a neck collar to prevent head movements^24^, ^30^, ^31^.

Clinical experience suggested that regardless of the repositioning maneuver or the chosen habituation technique^32^, ^33^ and the use or not of the bone vibrator on the mastoid^34^, treatment is effective in about 70 to 90% of the cases^20^, and this percentage is not affected by patients' age^35^. Currently, those restrictions after repositioning measures were questioned, requiring studies that compared groups submitted to canalith repositioning maneuver (Epley) and liberatory maneuver (Semont) with and without instructions, but no differences were observed between the two studied groups^29^, ^36^, ^37^, ^38^. Such studies were conducted to minimize the discomfort caused by the restrictions imposed on patients after the conduction of the procedure.

## OBJECTIVE

To assess the influence of posture guidance in early progression of patients with diagnosis of BPPV of posterior semicircular canal submitted to Epley maneuver.

## MATERIALS AND METHODS

The present study was a randomized prospective study that comprised the period between January 2003 and June 2004. The protocol of investigations followed all ethical rules in place at Hospital das Clínicas, FMUSP.

The sample comprised 50 patients diagnosed as having posterior semicircular canal BPPV.

Inclusion criteria:

- Positive Dix-Hallpike test;
- Agreed to participate in the study.

Exclusion criteria:

- Presence of neck restrictions that prevented conduction of Epley Maneuver;
- Use of antivertigo drugs.

Dix-Hallpike test was considered positive in case of triggering of dizziness and/or presence of horizontal-rotation nystagmus, clockwise to left canal and anti-clockwise to the right canal, in Rose's position with hyperextension and lateral neck rotation. Subjects that agreed to participate in the study were submitted to both consecutive canalith repositioning maneuvers at the same time, as proposed by Epley*.

After repositioning, patients were randomized to two groups:

Control group, who was not instructed.

**Figure 1:**
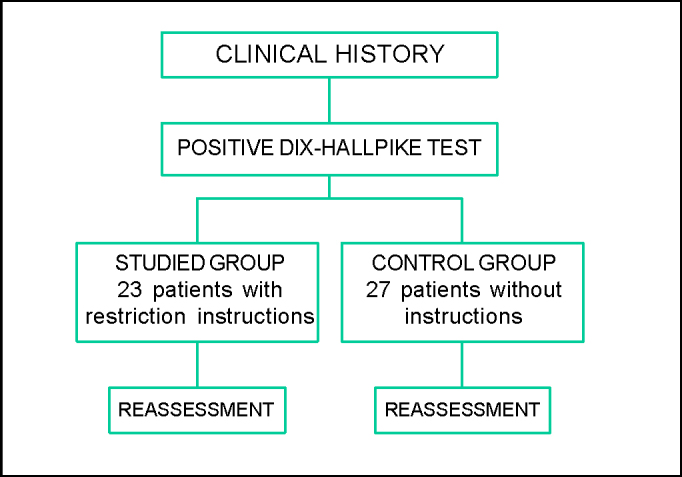
Sequential flow chart of the research method used.

Studied group, who received post-maneuver instructions: sleep with high-positioned head using two pillows; not to perform sudden head movement, especially looking to the sides, up or down; not to sleep over the affected ear. The study flow chart can be seen in Figure 1.

Patients were reassessed 72 +/- 24 hours after and divided into two groups: asymptomatic and symptomatic. The criteria for improvement were exclusion of main complaint (asymptomatic) and absence of dizziness and/or nystagmus in Dix-Hallpike test. We considered symptomatic the patients that reported partial improvement or absence of improvement after the maneuver and presented positive test result, even if in smaller amounts.

### Statistical Assessment

The variables in the study were gender, age, and presence of residual symptoms associated with positive result of Dix-Hallpike test. We used Chi-square test to assess the collected results. The level of significance was 0.05, as advocated for biological trials.

## RESULTS

We analyzed 50 patients, 16 male and 34 female. Mean age of the studied population was 60.94 +/- 15.30 years. As to laterality of affected canals, we found 29 cases of left posterior canals, and 21 right posterior canals.

The results obtained after the maneuvers of repositioning in relation to symptoms, gender and age of patients may be observed in Tables 1, 2 and 3.

**Table 1:** Final results obtained in post-maneuver assessment in both studied groups.

	asymptomatic	symptomatic	Total
with instructions	18	5	23
without instructions	17	10	27
Total	35	15	50

**Table 2:** Final results obtained in post-maneuver assessment classified by gender.

	asymptomatic	symptomatic	total
Female	25	9	34
Male	9	7	16
Total	34	16	50

**Table 3:** Final results obtained in post-maneuver assessment classified by age.

	asymptomatic	symptomatic	total
Up to 40 years	1	2	3
40-60 years	13	3	16
60-70 years	12	4	16
> de 70 years	11	4	15
Total	46	13	50

The difference of results between the group that was instructed and the group without instruction was not statistically significant (χ^2^ = 0.97).

The observed evolution when comparing male and female genders did not present statistically significant difference (χ^2^ = 0.80).

When assessing post-maneuver progression considering the different age ranges, we did not find statistically significant difference with the adopted value of p = 0.05 (χ^2^ = 2.49).

## DISCUSSION

According to our results, 37 (70%) of the 50 patients became asymptomatic after Epley maneuver when assessed within 72 ± 24 hours after conduction of the procedure. Our data are in accordance with the literature, whose references point towards success rate of the maneuver in about 60% to 100% of the cases as an effective treatment approach of BPPV^21^, ^28^, ^39^, ^40^

As to gender distribution, we observed predominance of female subjects (68%), which is also in accordance with literature reports^2^, ^42^. We should bear in mind, however, that even though BPPV prevalence studies report female predominance, when we consider younger age ranges, in which the main etiology is head trauma, the correlation between genders has no difference^4^. Despite clear female predominance, when considering clinical progression to treatment response, there were no statistically significant differences between genders.

The mean age of the studied population was 60.94 years and 31 (62%) of the patients in the sample were aged over 60 years, as previous reports on prevalence and incidence of BPPV have demonstrated^2^, ^3^. In the elderly, the diagnosis of BPPV is more frequent owing to associated co-morbidities^8^. In this age range, the disease normally has higher morbidity when associated with falls, which represents added risk to health and increased risk of mortality^40^, ^41^. It is interesting to notice that even though the elderly are more exposed to the disease, we can observe that efficacy of repositioning maneuver is the same as the one observed in other age ranges, in accordance with previous reports^35^.

An interesting fact in this study was that only 33% of the younger patients, up to the age of 40 years, responded well to the procedure, differently from other subjects in the sample that reached improvement rate over 75%. This isolated piece of data, even though not statistically significant, may be associated with etiology of the disease or still to the small sample in this age range, which comprised only three subjects. All these subjects presented history of head trauma that had triggered BPPV.

Even considering the possibility of self-resolution when treated with canalith repositioning maneuver, BPPV progresses better in the first month after the procedure, a fact that benefits patients and minimizes the duration of symptomatology^1^, ^18^, ^25^, ^28^, ^40^, ^43^. However, the performance of the maneuver does not show substantial benefits in the long-term follow-up, between 3 and 6 months, nor in relation to the possibility of recurrence, which seems to be more related with etiology of vestibular affections that are associated with BPPV than to the symptomatic treatment adopted^18^, ^44^, ^45^. It seems clear that canalith repositioning is the recommended treatment in cases of BPPV, but the impact of instructions on posture restriction after the maneuver is still a controversial issue^44^. Upon describing his maneuver, Epley^21^ recommended, in addition to the use of bone vibrator, some posture restrictions and head movements after repositioning procedure. Among the care recommended, he suggested sleeping in a seated position or with 45^o^ elevation of the head for 48 hours after the maneuvers. Other authors^27^, ^28^, who were stricter, recommended elevated head for 7 days after the maneuver, period in which sudden head movements and those that triggered dizziness would be avoided; not to sleep over the affected ear, and not to look up or down^29^. There were still others that instructed patients to wear a neck collar to prevent head movements^24^, ^30^, ^31^, a procedure not followed in our service owing to previous studies that suggested that neck collar and bone vibrator did not interfere in the outcomes of treatment^5^, ^44^.

All propositions, even though based on anatomical-physiological principles, make BPPV treatment a very disturbing approach to patients, who should take positions that they are not normally used to having, causing sensible affection to their quality of life. We should also consider that after the period of restrictions, patients may manifest great anxiety in view of the possibility of normally moving the head or sleeping over the affected the side to prevent symptomatology from restarting^38^. These facts potentially affect the elderly, which is known to be more fragile than younger people^8^ and present subsequent muscle pain when assuming any posture modification forced by the physician. We should bear in mind that the elderly population is significantly more affected by BPPV.

To get to know about the validity of post-maneuver instructions in relieving patients submitted to canalith repositioning, we randomized two groups and submitted them to posture restrictions recommended in the literature and did not give any instructions to the other group; we did not observe differences in the early clinical evolution of patients. The results are in accordance with those reported by other authors^29^, ^36^, ^38^ who did not detected better clinical evolution in patients submitted to different postures.

Concluding, treatment of BPPV with canalith repositioning maneuver is a simple option with satisfactory outcomes, regardless of posture restrictions traditionally recommended. The real etiology of BPPV is still questionable, but it seems clear that the initial proposition that repositioned particles would tend to get back to the posterior semicircular canal as a response to the position of the head is debatable. Only time and conduction of studies on the topic will clarify these facts. To present, it seems to be clear that there is no reason to submit our patients to these very uncomfortable recommendations.

## CONCLUSION

Our study suggested that Epley maneuver is an option for effective and safe treatment in 70% of the cases of BPPV approaching the posterior semicircular canal and posture restriction s post-maneuver studied in the present paper do not interfere in early progression of patients concerning symptomatology.
